# The Toxic Effects of Ppz1 Overexpression Involve Nha1-Mediated Deregulation of K^+^ and H^+^ Homeostasis

**DOI:** 10.3390/jof7121010

**Published:** 2021-11-25

**Authors:** Marcel Albacar, Lenka Sacka, Carlos Calafí, Diego Velázquez, Antonio Casamayor, Joaquín Ariño, Olga Zimmermannova

**Affiliations:** 1 Institut de Biotecnologia i Biomedicina & Departament de Bioquímica i Biologia Molecular, Universitat Autònoma de Barcelona, 08193 Cerdanyola del Vallès, Spain; Marcel.Albacar@uab.cat (M.A.); ccalafi1804@gmail.com (C.C.); Antonio.Casamayor@uab.cat (A.C.); 2 Laboratory of Membrane Transport, Institute of Physiology of the Czech Academy of Sciences, 142220 Prague, Czech Republic; lenkaptacnikova@seznam.cz (L.S.); Diego.VelazquezSanchez@fgu.cas.cz (D.V.)

**Keywords:** Ppz1 phosphatase, cation homeostasis, Nha1, intracellular pH, K^+^ transport, *Saccharomyces cerevisiae*

## Abstract

The alteration of the fine-tuned balance of phospho/dephosphorylation reactions in the cell often results in functional disturbance. In the yeast *Saccharomyces cerevisiae*, the overexpression of Ser/Thr phosphatase Ppz1 drastically blocks cell proliferation, with a profound change in the transcriptomic and phosphoproteomic profiles. While the deleterious effect on growth likely derives from the alteration of multiple targets, the precise mechanisms are still obscure. Ppz1 is a negative effector of potassium influx. However, we show that the toxic effect of Ppz1 overexpression is unrelated to the Trk1/2 high-affinity potassium importers. Cells overexpressing Ppz1 exhibit decreased K^+^ content, increased cytosolic acidification, and fail to properly acidify the medium. These effects, as well as the growth defect, are counteracted by the deletion of *NHA1* gene, which encodes a plasma membrane Na^+^, K^+^/H^+^ antiporter. The beneficial effect of a lack of Nha1 on the growth vanishes as the pH of the medium approaches neutrality, is not eliminated by the expression of two non-functional Nha1 variants (D145N or D177N), and is exacerbated by a hyperactive Nha1 version (S481A). All our results show that high levels of Ppz1 overactivate Nha1, leading to an excessive entry of H^+^ and efflux of K^+^, which is detrimental for growth.

## 1. Introduction

*Saccharomyces cerevisiae* Ppz1 is a type 1-related Ser/Thr protein phosphatase (692-aminoacid residues) composed of a C-terminal catalytic domain and a long N-terminal extension of about 350 residues [1,2]. In contrast to Glc7, which represents the ubiquitous PP1c, PPZ enzymes are restricted to fungi [3,4]. Ppz1 is regulated by two inhibitory subunits, Hal3 and Vhs3 [5,6,7], Hal3 being the more relevant inhibitor in vivo. These are moonlighting proteins since, in addition to their role in Ppz1 inhibition, they associate with Cab3, forming an unusual heterotrimeric phosphopantothenoylcysteine decarboxylase (PPCDC) involved in the biosynthesis of coenzyme A [7,8,9].

Early evidence demonstrated that the overexpression of Ppz1 is toxic for the yeast cell [5,10]. More recently, *PPZ1* was established as the gene for which the cell has the lowest tolerance limit [11], suggesting that it is the most toxic protein when overexpressed in budding yeast. The overexpression of Ppz1 blocks the cell cycle at the G_1_/S transition step [12], while more recent contributions have proven that the toxic effect is indeed caused by an increase in phosphatase activity and have provided a wealth of information about the cellular effects of Ppz1 overexpression. For instance, it has been shown that the excess of the phosphatase likely impairs protein translation, resulting in a Gcn2-mediated increase in the phosphorylation of the translation initiation factor eIF2α [13]. In addition, transcriptomic and phosphoproteomic profiling of Ppz1-overexpressing cells revealed profound changes in the transcriptome and identified more than 150 phosphoproteins with an altered phosphorylation pattern (most of them by dephosphorylation) at one or more phosphosites [14]. These experiments demonstrated that an excess of Ppz1 activity could perturb multiple targets and signaling pathways. Because Ppz1 has been recognized as a virulence factor in important human pathogenic fungi, such as *Candida albicans* [15] and *Aspergillus fumigatus* [16], as well as in the plant pathogen *Colletotrichum gloeosporioides* [17], and this protein is not present in animals or plants, it has been proposed as a possible antifungal target. Therefore, unraveling the specific targets affected by an excess of Ppz1 would be relevant from the point of view of both basic and applied science. However, the reasons for the toxicity of higher-than-normal levels of Ppz1 activity are not completely understood.

Ppz1 plays a key role in monovalent cation homeostasis, and it was demonstrated long ago that the deletion of *PPZ1* results in an increased salt tolerance [18]. This effect has been ascribed to the role of Ppz1 in two different processes: (i) the inhibition of potassium uptake through the K^+^ high-affinity Trk1 and Trk2 transporters, and (ii) a repressive effect on the expression of the Ena1 Na^+^, K^+^-ATPase involved in the response to salt stress [18,19,20,21]. The augmented K^+^ uptake in *ppz1∆* cells, leading to increased turgor pressure, was proposed to explain the sensitivity of this strain to cell wall damage conditions [2,5,18,22]. Therefore, a reasonable hypothesis to explain Ppz1 toxicity was that high Ppz1 levels would block Trk1/2-mediated K^+^ uptake, a situation incompatible with cell proliferation under standard growth conditions.

Another transport system involved in the maintenance of monovalent cation homeostasis is the plasma-membrane Nha1 antiporter, constitutively produced in cells in low amounts [23]. It is a housekeeping protein that actively exports Na^+^ and K^+^ in exchange for protons and, together with the main cation-efflux Ena ATPases, enables cell growth in the presence of high concentrations of alkali-metal-cation salts [23,24]. Due to its ability to export potassium cations, Nha1 also plays a key role in the internal pH and membrane potential regulation and in the immediate cell response to osmotic stress [25,26,27]. The transport activity of Nha1 is regulated via its long hydrophilic C-terminus (the last 554 residues, i.e., 56.2% of the whole 985-aa long protein) [23], whose presence is important for the maintenance of intracellular K^+^ content and cell cycle regulation [23,28]. A region of the Nha1 C-terminus rich in aspartyl and glutamic residues, amino acids 883–928, is necessary for maintaining maximal Na^+^ and Li^+^ transport activity [23,28], while amino acids 761–841 (C5 conserved domain) are important for the proper targeting of Nha1 to the plasma membrane [29]. Upon a hyperosmotic shock, the potassium-export activity of Nha1 transiently decreases to maintain a higher intracellular solute concentration [30]. In addition, the binding of 14-3-3 proteins to phosphorylated S481 at the beginning of the Nha1 C-terminus is apparently essential for the negative regulation of Nha1 activity under growth conditions, when cells need to accumulate high amounts of K^+^ [31].

We show here that increasing the external K^+^ concentration does not ameliorate the growth of Ppz1-overexpressing cells but that the deletion of the *NHA1* gene does. Further investigations allow us to postulate that high levels of Ppz1 hyperactivate Nha1, leading to an exacerbated entry of H^+^ and efflux of K^+^ that would be detrimental for growth. These effects would be blocked by the deletion of *NHA1*, thus explaining the growth improvement. Therefore, we must conclude that the overexpression of Ppz1 does alter monovalent cation homeostasis in a way that involves the plasma membrane Na^+^, K^+^/H^+^ antiporter Nha1.

## 2. Materials and Methods

### 2.1. Yeast Strains and Culture Media

Yeast cells were incubated at 28 °C or 30 °C in YP medium (1% yeast extract, 2% peptone), synthetic medium (YNB without amino acids; Difco, Sparks, MD, USA or Condalab, Madrid, Spain) lacking the appropriate selection requirements [32] or in translucent medium (YNB without amino acids, ammonium sulfate, and potassium; Formedium^TM^, Hunstanton, UK), which contains only traces of potassium [33,34]. The synthetic YNB w/o ammonium sulfate, folic acid, and riboflavin (YNB^MP^, MP Biomedicals, Solon, OH, USA) was used for intracellular pH measurements. The mixture of required auxotrophic supplements (the mixtures OMM or OMM-ura for cells transformed with plasmids) from a 50x concentrated sterile stock solution prepared according to another study [35] was added after media sterilization. Carbon sources, such as glucose (Glu, as in YPD), raffinose (Raff), or galactose (Gal), were added as indicated. Plates contained 2% or 3% agar.

Yeast strains used in this work derive from the wild-type BY4741 strain, with the exception of BW31 (a derivative of W303-1A), and they are described in Table 1. Strains CCP011 and ZCZ06 were made by the transformation of BY4741 and ZCZ01, respectively, with a 3.7 kbp *nha1::LEU2* cassette extracted with *Sac*I and *Xba*I from the plasmid pcRII-NHA1 (kindly provided by A. Rodríguez-Navarro). For intracellular pH measurements, strains were transformed either with the empty plasmid pVT100U or pHl-U, carrying the sequence of pHluorin [36].

For dot growth tests, strains were grown overnight in YPD or synthetic medium until saturation. Then, dilutions were prepared at OD_600_ = 0.05 plus two or three serial dilutions. From each dilution, 3 µL was loaded onto agar plates and incubated at 28 °C or 30 °C as indicated. For testing the growth rate at different pH, ZCZ01 and ZCZ06 cells were grown overnight in YPD until saturation. At this point, dilutions were prepared at OD_600_ = 0.004 in YP containing 2% raffinose and 1% galactose and buffered with 50 mM MES (pH 5.5 and 6.0) or HEPES (pH 6.5 and 7.0). Each dilution was distributed in triplicate on honeycomb plates (Thermo Fisher Scientific, Waltham, MA, USA), and cells were grown for two days at 28 °C with shaking in a Bioscreen C apparatus (Thermo Fisher Scientific, Waltham, MA, USA). The OD_600_ was monitored every 30 min.

### 2.2. Generation of Nha1 Variants

Two previously described multicopy plasmids, pNHA1-985 and pNHA1-985GFP, were used for the expression of the native Nha1 antiporter or its C-terminally-GFP-tagged version, respectively [23]. These plasmids were used as templates to introduce point mutations into the *NHA1* gene using a QuikChange XL Site-Directed Mutagenesis kit (Agilent Technologies, Santa Clara, CA, USA). For each mutation, two overlapping complementary oligonucleotides containing the corresponding nucleotide changes were designed (Table A1). The pNHA1-985 plasmid containing the *NHA1* allele with an S481A mutation was prepared previously [31]. Primers were purchased from Merck (Darmstadt, Germany). The accuracy of the mutation was confirmed by sequencing. Lasergene99 (DNASTAR Inc., Madison, WI, USA) was used for standard DNA and protein sequence analyses.

### 2.3. Media Acidification Tests

To analyze their H^+^ pumping ability, cells were grown on YPD until saturation. Then, cultures were diluted at OD_600_ = 0.2 with fresh YP plus 2% raffinose, and growth was resumed for 4–5 h until cultures reached OD_600_ = 0.6. Then, an aliquot was taken (t = 0). At this moment, galactose was added to the rest of the culture (2%) to initiate *PPZ1* expression. Further samples were taken at different time-points after galactose addition. Samples were immediately centrifuged and resuspended in 20 mL of YPD (so the final OD_600_ was 0.6 in all cases). Then, the cell suspension was supplemented with 20 mM KOH (final concentration) to increase the pH up to ≈8.0. Changes in pH were recorded online every 10 s for approximately 30 min using a Crison GLP21 pH-meter. Acidification values were calculated from the slope (nM H^+^/min) of the linear segment of the curve (min 8 to 30). No significant strain-specific differences in the number of cells during the collection of data were observed.

### 2.4. Preparation of Protein Extracts and Inmmunoblot

Procedures for protein extract preparation and immunoblot for Ppz1 detection were essentially as described in [13]. For the detection of Pma1 by immunoblot, cells were grown in YP plus 2% raffinose until OD_600_ = 0.6. A sample was taken at this point (t = 0), galactose was added to a final concentration of 2%, and further samples were acquired at different time-points. All samples (corresponding to 4.5 OD_600_) were processed as in a previous study [39]. Essentially, cultures were made 5% trichloroacetic acid, kept on ice for 15 min, and then centrifuged. Two washes with cold water were performed and pellets were stored at −80 °C. For SDS–PAGE analysis, pellets were resuspended with 120 μL of 2 × Laemmli buffer (preincubated at 37 °C). Five µL of each sample was loaded onto the gel, transferred to PVDF membranes (Immobilon-P, Millipore, Burlington, MA, USA), and probed with a rabbit polyclonal antibody against purified Pma1 (1:5000 dilution, a generous gift of Dr. R. Serrano), followed by incubation with a 1:15000 dilution of secondary anti-rabbit IgG-horseradish peroxidase antibody (GE Healthcare, Chicago, IL, USA). The 1:15000 dilution of anti-rabbit IgG-horseradish peroxidase secondary antibodies (GE Healthcare, Chicago, IL, USA). Immunoreactive proteins were detected using the ECL Prime Western blotting detection kit (GE Healthcare, Chicago, IL, USA) in a Versadoc 4000 MP imaging system (Bio-Rad, Hercules, CA, USA). Membranes were stained with Ponceau Red to monitor proper loading and transfer.

For the detection of Nha1-GFP, BW31 cells expressing native or various Nha1-GFP mutated versions from multicopy pNHA1-985GFP were grown in YNB plus 2% glucose to OD_600_ = 0.6, and the total extract of proteins was prepared as previously described [40]. Protein quantification was performed using the RDCD protein assay (Bio-Rad, Hercules, CA, USA). For SDS–PAGE (10% polyacrylamide), the amount corresponding to 120 µg of protein extracts was loaded for each sample and transferred to nitrocellulose membranes (Trans-Blot Turbo 0.2 µm Nitrocellulose) using a Trans-Blot Turbo Transfer System (Bio-Rad, Hercules, CA, USA). Membranes were incubated with a 1:500 dilution of monoclonal antibody against GFP (Roche, Basel, Switzerland). After that, a 1:10000 of secondary anti-mouse IgG-horseradish peroxidase (GE Healthcare) was used. Immunoreactive proteins were visualized with either the ECL Prime Western blotting detection kit (GE Healthcare, Chicago, IL, USA) or the Clarity Max Western ECL substrate kit (Bio-Rad, Hercules, CA, USA) in Versadoc or ChemiDoc imaging systems (Bio-Rad, Hercules, CA, USA). Membranes were stained with Ponceau Red to monitor proper loading and transfer.

### 2.5. Intracellular pH Measurements

For intracellular pH measurements, yeast strains expressing pHluorin were grown in YNB plus 2% raffinose medium to OD_600_ = 0.2, transferred into YNB^MP^ plus 2% raffinose to final OD_600_ = 0.180, and incubated for 2 h at 30 °C. Then, the fluorescence intensities were recorded using a Cytation 3 microplate reader (BioTek Instruments, Winooski, VT, USA; 100 μL of cells per well) equipped with monochromator optics (excitation wavelengths were 395 nm and 475 nm, emission 508 nm). The intensities of fluorescence at both excitation wavelengths were first read for 6 min (every 2 min, to set up a basal value of pH_in_), then 11 μL of H_2_O or 20% Gal (final concentration 2%) was added (t = 0), and the intensity of fluorescence was followed every 15 min for an additional 180 min. To eliminate the background fluorescence, a wild-type culture non-expressing pHluorin (transformed with empty vector pVT100U) was grown in parallel, and the corresponding values were subtracted from the fluorescence at each excitation wavelength (software Gen 5, BioTek Instruments, Winooski, VT, USA). The ratio of emission intensity I_395 nm_/I_475 nm_ at each time-point was used to calculate the intracellular pH according to the calibration curve prepared as described previously [41]. Four technical replicates per experiment were carried out for each set of conditions and strain. Presented data are means ± SEM of at least five experiments.

### 2.6. Potassium Content and Efflux Measurements

For measurements of K^+^ content, cells were grown in 80 mL YNB plus 2% raffinose medium to the early exponential phase (OD_600_ ~ 0.180), divided into two aliquots of 40 mL, incubated at 30 °C, and 4.5 mL H_2_O or 4.5 mL of 20% Gal (final concentration 2%) was added, respectively. Samples of cells (1 mL) were withdrawn at regular time intervals for 180 min, collected on Millipore membrane filters (Merck-Millipore, Co.Cork, Ireland), washed 3 times with 20 mM MgCl_2_, acid extracted, and the concentration of K^+^ in extracts was estimated by atomic absorption spectrophotometry as described previously [23]. Data shown are the means ± SEM of at least four replicates.

When the K^+^ efflux activity of Nha1 was measured, cells were grown in 70 mL YNB plus 2% raffinose medium to the early exponential phase (OD_600_ ~ 0.180), divided into two aliquots of 30 mL, and 3 mL H_2_O or 3 mL of 20% Gal (final concentration 2%) was added, respectively. After 1 h of incubation at 30 °C, cells were harvested, washed with cold water, and resuspended in a buffer of pH 4.5 consisting of 10 mM Tris, 0.1 mM MgCl_2_, and 2% glucose (the pH was adjusted to 4.4 with citric acid and Ca(OH)_2_ was added to increase the pH up to 4.5) and supplemented with 10 mM RbCl to prevent K^+^ reuptake. Cell samples were withdrawn at intervals during 40 min, and the intracellular concentration of K^+^ was estimated as above. Data shown are the means ± SEM of three replicates.

### 2.7. Fluorescence Microscopy

Microscopic images of yeast cells were acquired with an Olympus BX53 microscope with an Olympus DP73 camera. A Cool LED light source with 460 nm excitation and 515 nm emission was used to visualize Nha1 variants tagged with GFP. Cells containing the corresponding multicopy plasmids were grown overnight in YNB and observed when they reached the early exponential phase (OD_600_ ~ 0.2–0.3). Nomarski optics was used for whole-cell pictures.

### 2.8. Statistics

Data were analyzed with Microsoft Excel software 2016, and *p*-values were calculated using the two-tailed Student’s *t*-test.

## 3. Results

### 3.1. The Toxic Effects of Ppz1 Overexpression Are Supressed by Deletion of the NHA1 Antiporter Gene but Not by Potassium Supplementation

**Figure 1:**
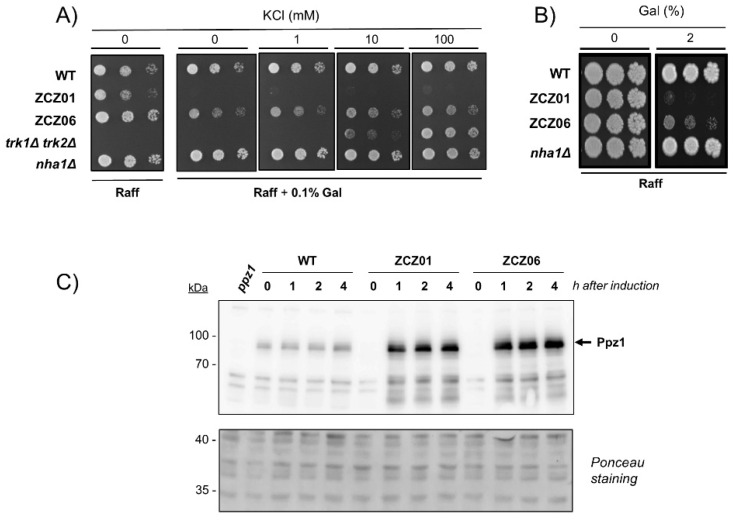
Deletion of *NHA1* counteracts Ppz1 overexpression toxicity. (**A**) Growth of strains BY4741 (WT), ZCZ01 (*GAL1*:*PPZ1*), ZCZ06 (*GAL1*:*PPZ1*, *nha1∆*), DSC32 (*trk1∆* *trk2∆*), and CCP011 (*nha1∆*) under the conditions of Ppz1 overexpression and in the presence of KCl. Cells were spotted on translucent medium with 2% raffinose and 0.1% galactose (where indicated) and supplemented with different concentrations of KCl. Plates were incubated for 6 days at 28 °C. (**B**) Cells were spotted as above in YP–Raff medium with or without 2% galactose (Gal). Growth was monitored after 6 days. (**C**) Immunodetection of Ppz1 in strains: BY4741 (WT), ZCZ01 (*GAL1*:*PPZ1*), and ZCZ06 (*GAL1*:*PPZ1 nha1∆*). Extracts from cells transferred to galactose-containing medium were prepared as described in Material and Methods, subjected to SDS–PAGE (10% gels), and transferred to membranes. Ppz1 was detected with a polyclonal antibody. Membranes were subjected to Ponceau red staining to monitor loading and transfer efficiency.

Because of the known effect of Ppz1 on potassium transport, we considered the possibility that the growth defect of Ppz1-overexpressing cells could be caused by insufficient potassium influx. However, supplementation of the medium with potassium up to levels that allow robust growth of strain DSC32 (*trk1∆* *trk2∆*, Table 1), totally deficient in high-affinity potassium transport, did not improve the growth of the ZCZ01 strain in which *PPZ1* is expressed at its own chromosomal location from the powerful *GAL1-10* promoter (Figure 1A). However, during a search of genes related to cation homeostasis that could influence the growth of strain ZCZ01, we observed that the deletion of *NHA1* (leading to strain ZCZ06) had a significant positive effect on growth (Figure 1A) even under conditions of strong overexpression of the phosphatase (2% galactose present in the medium, Figure 1B). Because the loss of toxicity could be due to a putative effect of the *nha1* deletion in the expression levels of Ppz1, we tested the amounts of the phosphatase by immunoblot analysis. As shown in Figure 1C, Ppz1 is already strongly expressed after one hour of galactose addition to the medium. These high levels are maintained for at least four hours and are not affected at all by the deletion of the antiporter gene. Therefore, the absence of Nha1 should be beneficial to Ppz1-overxpressing cells because it counteracts specific alteration(s) of cellular processes induced by high levels of the phosphatase.

### 3.2. Cells Overexpressing Ppz1 Show Noticiable Loss of Intracellular Potassium Levels and Increased Intracellular Acidification

**Figure 2:**
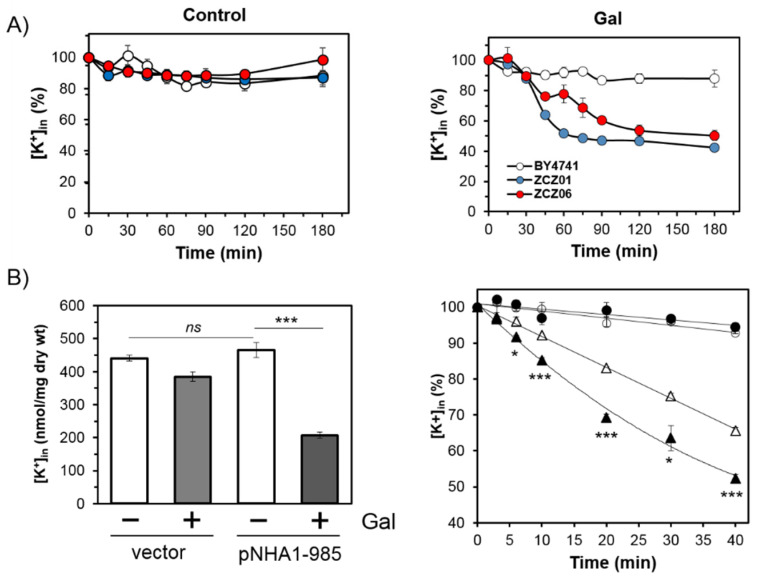
Overexpression of Ppz1 results in sharp decrease in intracellular potassium content and augmented Nha1-mediated K^+^ efflux. (**A**) Time course of changes of intracellular K^+^ content in strains BY4741 (wild-type; white), ZCZ01 (*GAL1*:*PPZ1*; blue), and ZCZ06 (*GAL1*:*PPZ1 nha1Δ*; red) grown in YNB plus 2% Raff and supplemented at time 0 min with galactose to induce the overexpression of Ppz1 (2% final concentration; right panel) or with water (control; left panel). The average initial concentrations of K^+^ (taken as 100%) for all three strains were similar 618 ± 62 and 626 ± 84 nmol/mg dry weight under control or Gal-induced conditions, respectively. Statistics for this figure are offered as Supplementary Material (Table S1). (**B**) Determination of Nha1-mediated K^+^ efflux. ZCZ06 cells carrying the empty vector (circles), or the pNHA1-985 plasmid (triangles) were grown on raffinose and split in two aliquots. Control cells received water (white bars and symbols), and Ppz1 was induced by adding galactose up to 2% (dark bars and symbols). After one h of incubation, cells were processed for measurement of K^+^ efflux (c.f. Materials and Methods). Left panel, initial intracellular concentrations of K^+^ at t = 0 of the efflux determination (taken as 100% for right panel; significant difference is indicated by asterisks *** *p* < 0.001; ns = not significant). Right panel, changes in K^+^ content for the different strains and conditions. The results represent mean values obtained in three independent experiments ± SEM. Significant differences between cells expressing Nha1 are indicated with asterisks (*, *p* < 0.05; *** *p* < 0.001).

**Figure A1:**
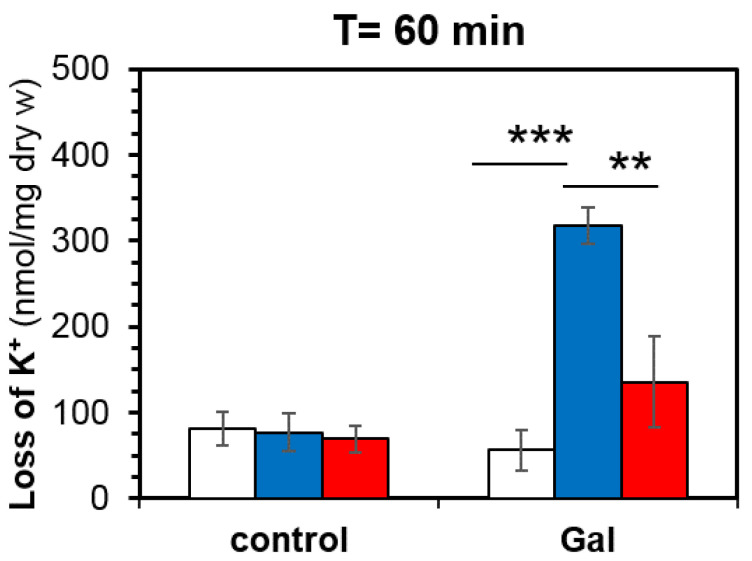
Amount of K^+^ lost from cells within the first 60 min of the experiment shown in Figure 2A. Strains are BY4741 (white), ZCZ01 (blue), and ZCZ06 (red). Data represent the mean ± SEM from at least four independent experiments, **, *p* < 0.01; ***, *p* < 0.001.

Since it is known that Nha1 has an important role in the maintenance of the proper intracellular levels of potassium and pH maintenance [42], we determined the intracellular potassium content in wild-type cells and in cells overexpressing Ppz1 in the presence or the absence of the *NHA1* gene. The initial concentration of K^+^ was not significantly different among the strains (on average, 618 ± 62 and 626 ± 84 nmol/mg dry weight for the control and induced cells, respectively). As shown in Figure 2A (right panel), the ZCZ01 cells exhibited a sharp decrease in their K^+^ content (up to 50% in the first 60 min; see also Appendix A Figure A1), and the levels were steadily low for at least 2 more hours. The lack of Nha1 did not affect the levels of potassium under standard circumstances, which were around 600 nmol/mg dry weight during the entire experiment. However, it clearly diminished the initial loss of K^+^ observed 30 min after the addition of galactose (Figure 2A—right panel, and Appendix A Figure A1), although the intracellular levels of the cation were also lower than those of the wild-type cells at the end of the experiment (Figure 2A—right panel).

We then monitored the efflux of potassium in cells overexpressing Ppz1. To this end, strain ZCZ06 was transformed with an empty plasmid or the same plasmid carrying the native *NHA1* gene. After one h of Ppz1 induction, the intracellular concentration of K^+^ in the ZCZ06 cells expressing Nha1 was about a half of the K^+^ in the same cells incubated in water (Figure 2B, left panel). This is a decrease similar to that observed for the ZCZ01 strain (Figure 2A, right panel). As observed in Figure 2B (right panel), K^+^ efflux is almost nil in the cells lacking Nha1, but the overexpression of Ppz1 clearly increases the efflux of the cation in cells where Nha1 is present.

**Figure 3:**
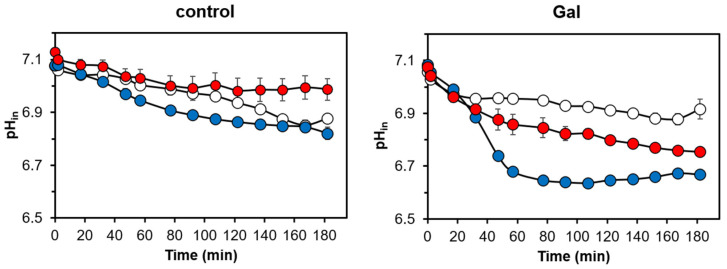
The absence of Nha1 decreases the intracellular pH drop caused by Ppz1 overexpression. Time course of intracellular pH changes in strains BY4741 (wild-type; white), ZCZ01 (*GAL1*:*PPZ1*; blue), and ZCZ06 (*GAL1*:*PPZ1 nha1∆*; red) expressing pHluorin and grown in YNB^MP^ plus 2% Raff. Galactose (2% final concentration; right panel) or water (control; left panel) were added at time 0. Data represent the mean ± SEM from at least 5 independent experiments (with four technical replicates each). Statistics for this figure are offered as Supplementary Material (Table S1).

We then investigated if the overexpression of Ppz1 was accompanied by changes in the intracellular pH. For this purpose, wild-type ZCZ01 and ZCZ06 cells were transformed with a vector expressing a pH-sensitive fluorescent protein (pHluorin). As shown in Figure 3, the addition of galactose resulted in a pronounced and sharp decrease in the intracellular pH in the ZCZ01 cells (around 0.4 units), whereas it had very little effect on the wild-type cells. The deletion of *NHA1* in the Ppz1-overexpressing cells (strain ZCZ06) markedly prevented the initial intracellular acidification, and the intracellular pH remained higher than that of strain ZCZ01 until the end of the experiment.

**Figure 4:**
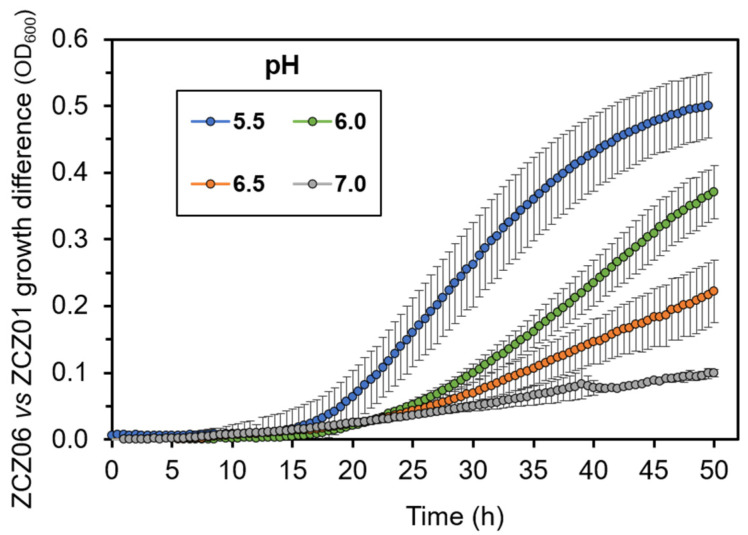
The growth improvement resulting from *NHA1* deletion in Ppz1-overexpressing cells is diminished with an increase in external pH. The growth rate of strains ZCZ01(*GAL1*:*PPZ1*) and ZCZ06 (*GAL1*:*PPZ1 nha1∆*) was compared in liquid medium buffered at different pH levels under conditions of Ppz1 overexpression (1% galactose) and the difference in growth (measured as OD_600_ values) calculated and plotted. Data are mean ± SEM from three independent experiments (with three technical replicas).

A possible explanation for the drop in the intracellular pH upon Ppz1 overexpression could be that it derives, at least in part, from a H^+^ import process (likely coupled with K^+^ efflux) mediated by Nha1. This would explain the fact that the absence of Nha1 attenuated intracellular acidification. We considered that such transport would be dependent on the H^+^ gradient across the plasma membrane and, consequently, the beneficial effect of the *nha1* deletion would be less evident when the external concentration of H^+^ decreases. To test this hypothesis, we grew ZCZ01 and ZCZ06 in media buffered at different pH levels (from 5.5 to neutrality) under conditions of Ppz1 overexpression and determined the difference in the growth between both strains. As documented in Figure 4, the progressive increase in the external pH led to the parallel attenuation of the effect of the *nha1* deletion. This beneficial effect became virtually nil when the external pH was 7.0.

### 3.3. Functionally Impaired Versions of Nha1 Do Not Counteract the Effect of Nha1 Loss in Ppz1-Overexpressing Cells

**Figure 5:**
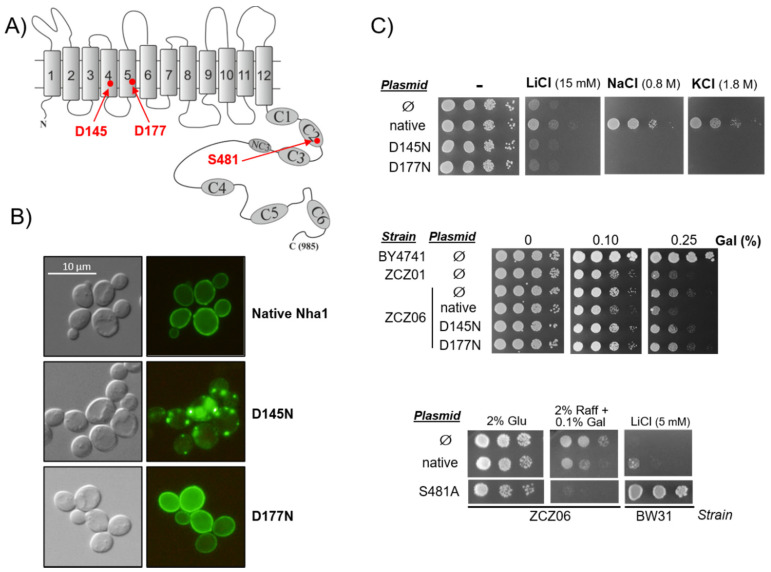
Functionally impaired versions of Nha1 counteract Ppz1 overexpression toxicity. (**A**) Schematic model of Nha1 structure with 12 TMS and 7 conserved domains in the hydrophilic C-terminus [29,44]. The positions of the three residues tested are highlighted. (**B**) Nomarski (left) and fluorescence (right) micrographs of BW31 cells expressing either native or mutated GFP-tagged Nha1 antiporters. Cells were grown in YNB plus 2% glucose to the early exponential phase. (**C**) Growth of BW31 (upper and lower panel) or ZCZ06 (middle and lower panel) cells containing either empty vector (∅) or expressing mutated versions of Nha1 on YNB plus 2% glucose (Glu) in the presence of salts or YNB plus 2% raffinose (Raff) and the indicated amount of galactose (Gal), respectively. In the middle panel, BY4741 and ZCZ01 (*GAL1*:*PPZ1*) transformed with an empty vector were used as references. Plates were incubated at 28 °C or 30 °C for two to five days.

The experiment described above suggests that the observed effects depend on the cation transport activity of Nha1. To further test this hypothesis, we prepared two different versions of Nha1, one with the D145N mutation and the other with the D177N substitution. Both mutations should result in the absence of an Nha1-mediated efflux of cations from the cells. The former mutation affects TM4 (Figure 5A) and results in the Nha1 mislocalization (absence in the plasma membrane) [43], while the latter Asp residue is located in TM5 (Figure 5A) and was predicted to be a cation binding site according to our structural models of yeast plasma-membrane Na^+^/H^+^ antiporters [44].

**Figure A2:**
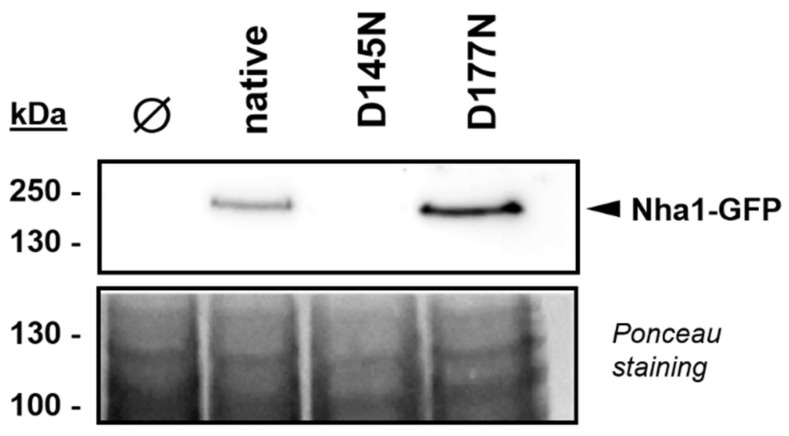
Immunodetection of Nha1-GFP versions. Extracts from BW31 cells expressing Nha1-GFP versions from multicopy plasmids growing in YNB plus 2% glucose were prepared as indicated in Material and Methods, subjected to SDS–PAGE (10% gel), and transferred to a nitrocellulose membrane. The Nha1-GFP was detected with a monoclonal anti-GFP antibody. The membrane was stained with Ponceau red to monitor loading and transfer efficiency.

Figure 5B shows the analysis of the cells expressing GFP-tagged versions of wild-type Nha1 or its D145N or D177N variants by fluorescence microscopy. As can be seen, the D145N mutation caused Nha1 to mislocalize in intracellular compartments. In contrast, the D177N variant was properly targeted in the plasma membrane, likely as the native Nha1. The immunoblot analysis of the cellular levels of the different proteins (Appendix A Figure A2) indicates that D177N was even expressed at a slightly higher level than the native Nha1 protein, while this technique failed to detect the D145N variant (perhaps because of its abnormal localization and/or faster degradation). To confirm the non-functionality of these mutated versions, we next tested their ability to provide cells with salt tolerance in comparison with the native Nha1. When expressed in the hypersensitive strain BW31 (which lacks both Ena ATPase and Nha1), neither the D145N nor the D177N variant were able to increase the cell tolerance to the Na^+^, Li^+^, or K^+^ cations, indicating that they could not mediate the efflux of alkali-metal cations from cells such as the native Nha1 (Figure 5C, upper panel). The expression of the native Nha1 protein in the ZCZ06 strain negatively affected the growth of the cells under Ppz1-overexpressing conditions (Figure 5C, middle panel) to a level similar to that of the ZCZ01 strain, as expected, since it ought to complement the *NHA1* deletion present in this strain. Remarkably, when the two Nha1 non-functional versions were introduced in the ZCZ06 cells, the cells grew in the presence of galactose as well as ZCZ06 transformed with the empty vector (Figure 5C, middle panel). In contrast, the mutation of S481A, which is known to significantly increase Nha1 cation efflux activity [31], strongly improved the growth of the BW31 strain in the presence of LiCl and dramatically blocked the growth of strain ZCZ06 (Figure 5C, bottom panel), suggesting that an excess of Nha1 activity is deleterious when Ppz1 levels are high. Taken together, these results reinforce the notion that the favorable effect of the *NHA1* deletion is due to the loss of the Nha1 cation/proton antiport capacity.

### 3.4. Overexpression of Ppz1 Limits the Capacity for Acidification of the Medium

**Figure 6:**
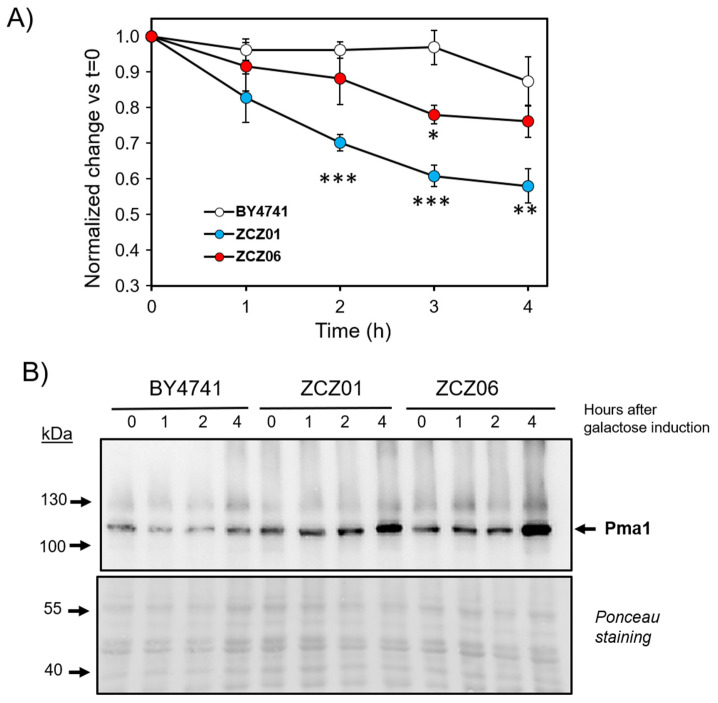
Overexpression of Ppz1 impairs the capacity for acidification of the medium. (**A**) Cultures of the indicated strains were prepared as described in Materials and Methods and supplemented with 2% galactose. Samples (20 mL, OD_600_ = 0.6) were taken at the indicated times, pH raised to 8.0 by the addition of KOH, and changes in pH were monitored for 30 min to calculate the acidification slope (nmols H^+^/min). The acidification capacity at t = 0 of the different strains was: BY4741, 0.231 ± 0.030; ZCZ01, 0.190 ± 0.025; and ZCZ06, 0.339 ± 0.009 nM H^+^/min). For plotting, data were adjusted to t = 0 and represent the mean ± SEM from at least 5 independent experiments. *, *p* < 0.05; **, *p* < 0.01; ***, p < 0.001 with respect to the BY4741 (WT) strain. (**B**) Protein extracts from these strains were prepared at different times after Ppz1 induction, resolved by SDS–PAGE (8% gel), blotted, and Pma1 detected with polyclonal antibodies. Ponceau staining of the membrane is shown at the bottom.

To further characterize the basis for the observed intracellular acidification, we tested the ability of the wild-type, ZCZ01, and ZCZ06 strains to release protons to the medium under standard conditions for Ppz1 overexpression. To this end, the pH of the culture was shifted to pH 8.0, and the rate of increase in the H^+^ concentration in the medium was followed for 30 min. We observed that, at time zero, the acidification capacity of the wild-type and ZCZ01 strains was not significantly different (0.231 ± 0.030 vs. 0.190 ± 0.025 nM H^+^/min), whereas that of strain ZCZ06 was slightly higher (0.339 ± 0.009 nM H^+^/min). However, as seen in Figure 6A, the ability of the ZCZ01 cells to acidify the medium was markedly lower than that of the wild-type strain, and the deletion of *NHA1* (strain ZCZ06) substantially normalized this behavior (the differences between ZCZ01 and ZCZ06 were significant at least at *p* < 0.05, except for t = 1 h).

The H^+^-ATPase Pma1 is a major contributor to the capacity of yeast cells to pump out protons. Because the overexpression of Ppz1 is known to induce a blockage in protein translation, we wondered whether the decrease in the acidification capacity of the ZCZ01 strain could be due to lower-than-normal levels of the Pma1 protein, as well as whether this could be affected by the deletion of *NHA1*. To test this possibility, we prepared protein extracts from wild-type, ZCZ01, and ZCZ06 cells and monitored the presence of the Pma1 protein by immunoblot. As shown in Figure 6B, the Pma1 levels were actually higher in ZCZ01 than in wild-type cells, and the amount of Pma1 in the ZCZ06 strains did not differ from that of ZCZ01. Therefore, we must conclude that the decreased acidification of the medium in the ZCZ01 cells is not caused by a reduction in the Pma1 protein levels.

## 4. Discussion

Recent work has provided evidence that the toxic effect of Ppz1 overexpression derives from the alteration of numerous cellular processes [13,14]. Because of the previously documented role of Ppz1 in the regulation of monovalent cation transport (mainly K^+^ and Na^+^) [18,19,20,21] and the importance of this homeostatic process for normal yeast proliferation [42], we considered that Na^+^/K^+^ homeostasis could be one of the cellular processes altered by Ppz1 overexpression. Ppz1 was defined long ago as a negative regulator of K^+^ influx through the high-affinity plasma-membrane transporters Trk1 and Trk2 [20,21,22]. Therefore, it was reasonable to assume that high levels of the phosphatase could result in a strong inhibition of K^+^ transport, which was known to severely affect growth. However, the fact that an increase in the amount of available K^+^ in the medium up to levels that allow normal growth of a *trk1∆* *trk2∆* strain (thereby devoid of high-affinity K^+^ transport) does not improve the growth of the Ppz1 overexpressing strain at all strongly argues against the hypothesis that the negative regulation of Trk1/Trk2 transporters contributes to Ppz1 toxicity. In this context, the remarkable observation that the elimination of the Nha1 antiporter in the ZCZ01 strain significantly ameliorates its growth under Ppz1-overexpressing conditions could be interpreted as monovalent cation homeostasis being somehow disturbed when high levels of Ppz1 are present in the cell and that such perturbation negatively affects cell growth.

Our observation that the accumulation of Ppz1 parallels with a sharp decrease in the intracellular concentration of K^+^ (Figure 2A and Appendix A Figure A1) and a dramatic acidification of the cytosol (Figure 3) confirms the impact of an excess of Ppz1 on cation homeostasis. Nha1 is a housekeeping protein that actively exports Na^+^ and K^+^ (and their toxic analogues Li^+^ and Rb^+^, respectively) in exchange for protons [45], thus playing an important role in the regulation of intracellular pH [27] and membrane potential [25]. The fact that the deletion of *NHA1* in cells overexpressing Ppz1 partially normalizes both K^+^ and H^+^ intracellular content indicates that Nha1′s function contributes to these changes. Therefore, we hypothesized that the excess of Ppz1 activity could lead to the hyperactivation of Nha1, resulting in an exacerbated influx of H^+^ in exchange for K^+^ ions. This scenario is supported by three observations. First, the K^+^ efflux activity of Nha1 in cells overexpressing Ppz1 was much higher than in cells in which the expression of Ppz1 was not induced (Figure 2B). Second, a decrease in the intra/extracellular pH gradient, which would lead to a decreased role of Nha1 in K^+^/H^+^ exchange, abolishes the beneficial effect of the Nha1 deletion (Figure 4). Third, the use of two non-functional versions of Nha1 (D145N and D177N) showed that neither of them could complement the *nha1* deletion in BW31 cells, and their expression in ZCZ06 cells led to the same phenotype that was observed when this strain was transformed with the empty vector (Figure 5C). The non-functionality of the Nha1 (D145N) version is most likely caused by mislocalization in the intracellular compartments, which is in agreement with a previous work of Mitsui and coworkers [43]. As shown in Figure 5C, the Nha1(D177N) version was unable to improve the salt tolerance of BW31 cells, although it was correctly targeted to the plasma membrane (Figure 5B) and expressed at high levels (Figure A2). This result constitutes experimental evidence for the importance of aspartate 177, predicted by modelling to be a cation binding site for Nha1′s transport function [44].

Under the conditions of Ppz1 overexpression, ZCZ06 cells carrying any of the non-functional Nha1 versions grew better than the cells expressing native Nha1 (Figure 5C). Therefore, it can be concluded that the toxicity of a high level of Ppz1, which is accompanied by a rapid loss of K^+^ (Figure 2) and a drop in intracellular pH (Figure 3), depends on the presence of Nha1 in the plasma membrane and its ability to mediate cation transport.

While Nha1 is expressed at a relatively constant level, its activity has been reported to be controlled by phospho/dephosphorylation reactions affecting its long C-terminal tail. The phosphorylation of T765 and T876 by the Hog1 kinase was proposed to be important for the short-term involvement of Nha1 in the response to salt stress [26], while serine 481 is responsible, when phosphorylated by an unknown kinase (different from Hog1), for binding to yeast 14-3-3 proteins, which decreases the Nha1 activity [31,46]. Importantly, the mutation S481A (which prevents its phosphorylation) significantly increases the cation efflux activity via Nha1 [31]. In this context, the observation that the ZCZ06 strain expressing the S481A Nha1 version actually grows worse than the ZCZ06 expressing the native Nha1 from the same multicopy plasmid (Figure 5C), as expected for a hyperactive Nha1 exchanger, provides further support for our proposal. It is worth noting our recent report that, under the same experimental setting used in this work, Nha1 became significantly dephosphorylated at Ser481 (>30%) as little as one hour after Ppz1 induction [14]. In addition, the fact that a physical interaction between Ppz1 and Nha1 was detected by Tarassov and coworkers [47] using a protein-fragment complementation assay could be a hint that Nha1 is a direct target for Ppz1-mediated dephosphorylation.

**Figure A3:**
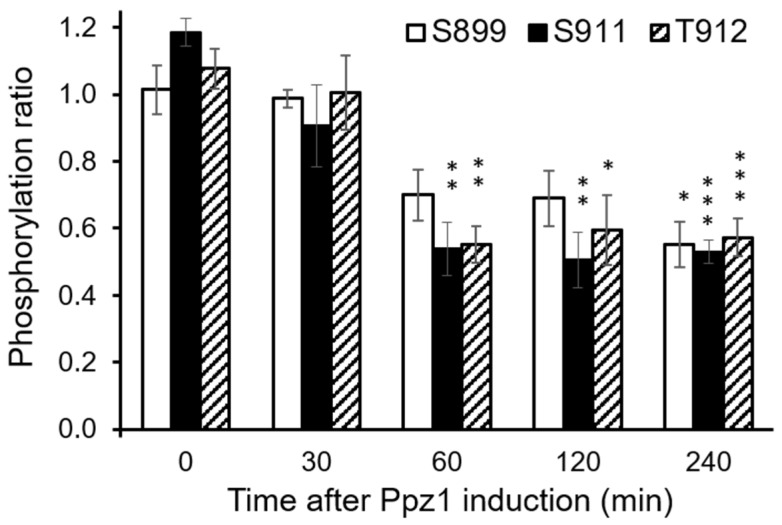
Changes in the phosphorylation state of Pma1 S899, S911, and T912 residues upon the overexpression of Ppz1. Data were extracted from reference [14] and are expressed as the ratio between Ppz1-overexpressing cells vs. wild-type cells (means ± SEM from four independent experiments). *, *p* < 0.05; **, *p* < 0.01; ***, *p* < 0.001.

We show here that the deletion of *NHA1* in a Ppz1-overexpressing strain only partially normalizes the growth rate and K^+^ and H^+^ intracellular contents. This suggests the existence of additional targets that are important for monovalent cation homeostasis and are affected by high Ppz1 levels. Among the different possibilities considered, the role of Ppz1 on *ENA1* could be discarded because Ppz1 does not actually act as an activator but as a repressor of its expression, which is very low in the absence of stress (in fact, Figure 2B shows that virtually no K^+^ efflux can be measured in ZCZ06 cells lacking *NHA1* but containing *ENA* genes). In yeast cells, the plasma membrane H^+^-ATPase Pma1 is the major determinant of plasma membrane potential as a result of its electrogenic transport of H^+^ and is believed to be a major determinant of cytosolic pH [48]. Our observations that cells overexpressing Ppz1 failed to properly acidify the medium, and that this limitation was only partially overcome by the deletion of *NHA1* (Figure 6A), suggest that Pma1′s function might be negatively affected by the excess of Ppz1. Pma1 activity can be controlled by reversible phosphorylation and by transcriptional modulation (i.e., in response to glucose; see [48] and references therein). Because high levels of Ppz1 are known to negatively affect translation [13], it was important to monitor the levels of the ATPase during the overexpression of the phosphatase since a decrease in the Pma1 levels could explain a loss in acidification capacity. However, our data indicate that the levels of Pma1 are actually higher in the cells expressing Ppz1 than in normal cells (Figure 6B). Pma1 is phosphorylated in the cell at 20 sites (at least), mostly located on its N- and C-terminal moieties (SGD at http://www.yeastgenome.org/, accessed date 05 October 2021). Among them, the phosphorylation of S911 and T912 appear to be important for the activation of Pma1, with a lesser contribution of the highly conserved serine S899 [49,50]. It is worth noting our recent work showing that the overexpression of Ppz1 affected the phosphorylation state of a limited number of Pma1 residues, and these included not only S911 and T912 but also S899 [14]. The decrease in all three cases was significant, with a reduction of up to one-half of the normal phosphorylation levels, already noticeable after 60 min of Ppz1 induction and sustained over time (see Appendix A Figure A3). Therefore, it is not unlikely that these changes in the phosphorylation state of Pma1 may account for a decrease in its H^+^ pumping capacity, thus explaining (at least in part) the Nha1-independent changes in the intracellular H^+^ content.

The activity of the Pma1 H^+^-ATPase is the major consumer of ATP in the cell, amounting to between 20 and 50% of the cellular ATP [51], and there is plenty of evidence that the pharmacological inhibition of Pma1 [52,53] or point mutations in the protein that lead to partial defects in the pumping activity [54] result in increased ATP levels. In this context, it is significative that, in parallel to the dephosphorylation of relevant Pma1 residues, we observed that the cells expressing high levels of Ppz1 accumulate higher than normal (up to two-fold) ATP levels [14]. Such an increase fits well with our proposal that Pma1 becomes inhibited when Ppz1 levels are abnormally high.

## Figures and Tables

**Table 1:** Yeast strains used in this work.

Strain	Genotype	Reference
BY4741	*MATa his3Δ1 leu2Δ0 met15Δ0 ura3Δ0*	EUROSCARF
ZCZ01	BY4741 p*GAL1-10*:*PPZ1*	[13]
ZCZ06	BY4741 p*GAL1-10*:*PPZ1 nha1::LEU2*	This work
DCS32	BY4741 *trk1*::*LEU2 trk2*::*nat1*	[37]
CCP011	BY4741 *nha1*::*LEU2*	This work
BW31	*MATa ena1*::*HlS3*::*ena4 nha1*::*LEU2*	[38]

## Data Availability

Data for construction of Appendix A Figure A3 can be found in Supplementary file 4 from our reference [14].

## Supplementary Materials

The following are available online at https://www.mdpi.com/article/10.3390/jof7121010/s1. Table S1, *p*-values for data presented in Figure 2A and Figure 3.

## Appendix A

**Table A1:** Oligonucleotides used in this work.

Name	Sequence *
NHA1_D145N_Fwd	5′-CTGCGTGCATTACCGCAACAAATCCTATTCTGGCGCAGTCGG-3′
NHA1_D145N_Rew	5′-CCGACTGCGCCAGAATAGGATTTGTTGCGGTAATGCACGCAG-3′
Nha1D177N-F	5‘-TCAGGCTGCAATAATGGTATGGCCTTTC-3‘
Nha1D177N-R	5‘-GAAAGGCCATACCATTATTGCAGCCTGA-3‘

* Underlined are nucleotides changed from the native *NHA1*.
